# Early Effects of HTLV-1 Infection on the Activation, Exhaustion, and Differentiation of T-Cells in Humanized NSG Mice

**DOI:** 10.3390/cells10102514

**Published:** 2021-09-23

**Authors:** Otávio de Melo Espíndola, Esther Siteur-van Rijnstra, Esmay Frankin, Kees Weijer, Yme Ubeles van der Velden, Ben Berkhout, Bianca Blom, Julien Villaudy

**Affiliations:** 1 Laboratory for Clinical Research in Neuroinfections, Evandro Chagas National Institute of Infectious Diseases, Oswaldo Cruz Foundation (FIOCRUZ), Rio de Janeiro 21040-900, Brazil; 2 Department of Experimental Immunology, Amsterdam University Medical Centers, Location AMC, University of Amsterdam, 1105 AZ Amsterdam, The Netherlands; e.siteurvanrijnstra@amsterdamumc.nl (E.S.-v.R.); esmayfrankin@live.nl (E.F.); kweijer@xs4all.nl (K.W.); b.blom@amsterdamumc.nl (B.B.); 3 Laboratory of Experimental Virology, Department of Medical Microbiology, Amsterdam University Medical Centers, Location AMC, University of Amsterdam, 1105 AZ Amsterdam, The Netherlands; y.u.vandervelden@amsterdamumc.nl (Y.U.v.d.V.); b.berkhout@amsterdamumc.nl (B.B.); julien@jnspreclinical.com (J.V.); 4 J&S Preclinical Solutions, 5345 RR Oss, The Netherlands

**Keywords:** HTLV-1, ATLL, humanized mice, NSG mice, T-cells, chemokine receptors, exhaustion

## Abstract

Adult T-cell leukemia/lymphoma (ATLL) is an aggressive malignancy of CD4^+^ T-cells associated with HTLV-1 infection. In this study, we used the model of immunodeficient NSG mice reconstituted with a functional human immune system (HIS) to investigate early events in HTLV-1 pathogenesis. Upon infection, human T-cells rapidly increased in the blood and lymphoid tissues, particularly CD4^+^CD25^+^ T-cells. Proliferation of CD4^+^ T-cells in the spleen and mesenteric lymph nodes (MLN) correlated with HTLV-1 proviral load and CD25 expression. In addition, splenomegaly, a common feature of ATLL in humans, was also observed. CD4^+^ and CD8^+^ T-cells predominantly displayed an effector memory phenotype (CD45RA^−^CCR7^−^) and expressed CXCR3 and CCR5 chemokine receptors, suggesting the polarization into a Th1 phenotype. Activated CD8^+^ T-cells expressed granzyme B and perforin; however, the interferon-γ response by these cells was limited, possibly due to elevated PD-1 expression and increased frequency of CD4^+^FoxP3^+^ regulatory T-cells in MLN. Thus, HTLV-1-infected HIS-NSG mice reproduced several characteristics of infection in humans, and it may be helpful to investigate ATLL-related events and to perform preclinical studies. Moreover, aspects of chronic infection were already present at early stages in this experimental model. Collectively, we suggest that HTLV-1 infection modulates host immune responses to favor viral persistence.

## 1. Introduction

Human T-lymphotropic virus type 1 (HTLV-1) infects 5–10 million individuals worldwide, with clusters of high endemicity in Japan, the Caribbean, the Middle East, sub-Saharan Africa, and South America [1]. HTLV-1 is the etiological agent of two life-threatening diseases: a malignancy of CD4^+^ T-cells termed adult T-cell leukemia/lymphoma (ATLL) [2,3], and an inflammatory disorder with progressive neurological disability and motor impairment known as HTLV-1-associated myelopathy/tropical spastic paraparesis (HAM/TSP) [4,5]. However, over 90% of infected individuals remain asymptomatic and the reasons why only a minority develop symptoms after decades of silent infection are not fully understood.

CD4^+^ and CD8^+^ T-cells are the main targets of HTLV-1 infection in vivo. These cells display a CD45RO^+^ effector/memory phenotype [6] with increased levels of activation markers such as HLA-DR, CD25, and CD69 [7,8,9]. The infection triggers a T helper type 1 (Th1) immune response with robust expression of interferon-γ (IFN-γ) and IFN-γ inducible genes [10,11], and a high frequency of HTLV-1-specific CD8^+^ T-cells [12,13]. However, HTLV-1 infection persists despite the humoral and cell-mediated responses, leading to chronic stimulation of immunological mechanisms. Two viral regulatory proteins are involved in this process: the transactivator (Tax) and the HTLV-1 basic leucine zipper (HBZ) proteins, which induce cell cycle and inhibit apoptosis of infected cells [14,15,16,17,18]. Tax also interferes with cellular DNA repair and the activity of the tumor suppressor proteins p53 and p16, thus enhancing the accumulation of mutations that promote the transformation of T-cells. On the other hand, HBZ is involved in viral persistence and immune escape, especially when ATLL is already established [19]. While HBZ is consistently expressed in ATLL cells [20], Tax expression is lost in approximately 60% of the cases [21].

Over the past decades, murine models have significantly contributed to the understanding of HTLV-1 pathogenesis. Transgenic mice expressing HTLV-1 Tax protein develop leukemia and lymphomas of T-cells, with histopathological findings resembling those of ATLL patients [22,23]. In turn, transgenic mice with CD4^+^ T-cell-restricted HBZ expression display an increased frequency of CD4^+^ T cells in the spleen, high frequency of CD4^+^FoxP3^+^ regulatory T-cells (Tregs), and chronic inflammation in the skin and lungs, as seen in HTLV-1-infected individuals [20,24]. Recently, the development of immunodeficient mice reconstituted with a functional human immune system (HIS) after the injection of human CD34^+^ hematopoietic stem cells (HSCs) has opened the door for a detailed in vivo investigation of HTLV-1 pathogenesis [25]. It was shown in HIS-BALB/c Rag2^−/−^IL-2Rγc^−/−^ (BRG) mice that HTLV-1 infection accelerates the thymic development and maturation of T-cells, particularly of CD4^+^ T-cells [26]. HIS-NOD.Cg-Prkdc^scid^Il2rg^tm1Wjl^/SzJ (NSG) mice reconstituted with human CD133^+^ HSCs developed leukemia and lymphomas upon HTLV-1 infection, and presented elevated serum levels of IL-6, IL-8, IL-10, IL-12, IL-13, IFN-γ, TNF-α, GM-CSF, and CCL4 [27]. Percher et al. [28] demonstrated that Tax-dependent secretion of leukotriene B4 by infected T-cells promotes the recruitment, cell–cell contact, and HTLV-1 propagation, and Pérès et al. [29] showed that the PDZ domain-binding motif of Tax sustains T-cell proliferation in HTLV-1-infected humanized mice. Furthermore, Vicario et al. [30] showed that fibroblasts play a role in secreting pro-inflammatory cytokines and chemokines, thus producing a microenvironment favorable for the tumorigenesis of the ATLL lymphoma type. However, it is still not clear how HTLV-1 infection impacts the composition of T-cell populations in secondary lymphoid tissues, and how the virus escapes from host immune responses to favor viral persistence. In this study, T-cells and myeloid dendritic cells (mDCs) from blood, spleen, and mesenteric lymph nodes (MLN) of HTLV-1-infected HIS-NSG mice were characterized to evaluate early events linked to establishment of chronic infection.

## 2. Materials and Methods

### 2.1. Ethics Statement

Human fetal liver tissue was obtained from elective abortions. Gestational age was determined by ultrasonic measurement of the skull diameter and ranged from 14 to 20 weeks. The use of fetal tissue was approved by the medical ethical committee of the Academic Medical Center (AMC) of the University of Amsterdam (UvA) (protocol number 03/038#03.17.0211), and it was contingent on informed consent. The study protocol was approved by the animal experimental committee Amsterdam (DEC) of AMC-UvA (protocol number DHL161), and all efforts were made to minimize animal suffering. 

### 2.2. Isolation of Hematopoietic Stem Cells from Human Fetal Liver Samples

This was performed accordingly to Legrand et al. [31,32]. Briefly, fetal liver tissue was disrupted, and the cell suspension was centrifuged on a density gradient with Lymphoprep™ (Axis Shield, Dundee, Scotland, UK). CD34^+^ HSCs were enriched using immunomagnetic beads following the manufacturer’s instructions (CD34 MicroBead Kit, Miltenyi Biotec, Bergisch Gladbach, Germany), and further purified into CD34^+^CD38^−^lineage^−^ (CD3/CD14/CD19/CD56/BDCA2) cells (Purity ≥ 99.0%) by fluorescence-activated cell sorting on a FACSAria II (Becton Dickinson, Franklin Lakes, NJ, USA). Cells were frozen in fetal bovine serum (FBS) containing 10% DMSO and kept in liquid nitrogen until transplant into newborn NSG mice.

### 2.3. Establishment of HIS-NSG Mice

HIS-NSG mice were generated by injecting human HSCs into five-day-old mice. Initially, newborn NSG mice were sub-lethally irradiated once (1 Gy) using a ^137^Cs source and human CD34^+^CD38^−^lineage^−^ cells (5 × 10^4^ cells) were intrahepatically injected. Eight weeks later, peripheral blood was collected from the submandibular vein to determine the reconstitution of a HIS, and mice displayed a median of 55.3% human leukocytes in blood (Interquartile range: 43.8% to 62.9%). The mice were housed in individually ventilated cage with sterile bedding, food, and acidified water ad libitum. They were maintained in a room with a 12 h–12 h light–dark cycle at a controlled temperature.

### 2.4. HTLV-1 Infection of HIS-NSG Mice and Processing of Samples

MT2 cells (RRID:CVCL_2631), an HTLV-1-infected T-cell line, were irradiated with 77 Gy from a ^137^Cs source in a IBL 637 γ-ray machine (CIS BIO international, Saclay, France,) at 1.28 Gy/min. This dose was previously shown to inhibit cell proliferation but not HTLV-1 production [26]. Twelve- to thirteen week-old HIS-NSG mice were intraperitoneally inoculated with 1 × 10^6^ γ-irradiated MT2 cells in 0.1 ml of phosphate buffer saline (PBS) or mock-infected with PBS only. Mice were euthanized after isoflurane anesthesia at 30 days and 60 days from inoculation, when blood, spleen, and MLN were collected. White blood cells were obtained by lysis of red blood cells using ammonium chloride solution, and then washed twice with PBS. Spleen and MLN were gently minced in RPMI 1640 medium with 10% FBS to obtain single-cell suspensions, and splenocytes were isolated by centrifugation on Lymphoprep™.

### 2.5. Monoclonal Antibodies and Flow Cytometry Assays

Staining of fresh cells for analysis by flow cytometry was performed with the following fluorophore-conjugated anti-human monoclonal antibodies (mAbs): CD3-BV785 (clone OKT3), CD8-APC (Clone RPA-T8), CD45-FITC (Clone HI30), CD45RA-PE (Clone HI100), CD80-BV421 (Clone 2D10), CD11c-PerCP/Cy5.5 (Clone Bu-15), CCR4 (CD194)-PE/Cy7 (Clone L291H4), CXCR3 (CD183)-APC/Cy7 (Clone G025H7), CCR7 (CD197)-PE/Cy7 (Clone G043H7), CD25-PE (Clone BC96) (all from BioLegend, San Diego, CA, USA); CD4-eFluor450 (Clone OKT4), CD14-APC/eFluor780 (Clone 61D3), HLA-DR-APC (Clone LN-3), CCR5 (CD195)-PE (Clone eBioT21/8), FoxP3-APC (Clone 236A/E7), Perforin-PE (Clone delta G9), PD-1 (CD279)-PE/Cy7 (Clone eBioJ105), IFN-γ-APC/eFluor780 (Clone 4S.B3) (all from eBioscience); CD86-BV650 (Clone FUN-1), Ki67-BUV395 (Clone B56), Granzyme B-BV421 (Clone GB11) (all from BD Biosciences, Franklin Lakes, NJ, USA). Staining of cell surface antigens was performed in PBS or RPMI 1640 medium containing 2% FBS. For staining of granzyme B, perforin and IFN-γ, 0.5–1 × 10^6^ cells were stimulated with phorbol-myristate-acetate (PMA) (50 ng/mL) and ionomycin (1µg/mL) in RPMI 1640 medium with 10% FBS for 6 h, and intracellular protein transport was inhibited with 1× GolgiStop™ (BD Biosciences, Franklin Lakes, NJ, USA) during the last 4 h of incubation. Intracellular staining was carried out with the FoxP3/Transcription Factor Staining Buffer Set (eBioscience, San Diego, CA, USA), according to the manufacturer’s instructions. Cells were fixed with 1% paraformaldehyde in PBS and 200.000 events were acquired using FACSDiva™ software in an LSRFortessa™ cell analyzer (BD Biosciences, Franklin Lakes, NJ, USA). FlowJo^®^ v10 was used for data analysis, which was performed after the exclusion of doublets by plotting FSC-H × FSC-A and the selection of human leukocytes by gating for CD45^+^ cells. 

### 2.6. Quantification of HTLV-1 Proviral Load (PVL)

DNA was extracted from peripheral blood leukocytes (PBL), splenocytes, and MLN cells with the NucleoSpin^®^ Blood kit (Macherey-Nagel, Düren, Germany), according to the manufacturer’s instructions. HTLV-1 PVL was determined by quantitative real-time PCR using the Rotor-Gene Probe PCR kit (Qiagen, Hilden, Germany), and 5′-FAM and 3′-TAMRA-labeled TaqMan^®^ probes (Sigma-Aldrich, St. Louis, MO, USA) for the detection of human *β-globin* and HTLV-1 *tax* genes in independent reactions [33]. Standard-curves for quantification of human *β-globin* and HTLV-1 *tax* genes were established with 10-fold serial dilutions of DNA from, respectively, normal human PBL, and the TARL-2 cell line (RRID:CVCL_0557), which carries a single copy of HTLV-1 provirus per cell. It was assumed that one nanogram of diploid human DNA contains 333 copies of the *β-globin* gene and one nanogram of TARL-2 DNA contains 167 copies of HTLV-1 *tax* gene. HTLV-1 PVL was calculated as *tax* copies/(*β-globin* copies/2), and the values were normalized by the frequency of human T-cells (CD45^+^CD3^+^ cells) determined in each sample by flow cytometry.

### 2.7. Statistical Analysis

Statistical analysis was performed using GraphPad Prism v.5 software. All analyses were carried out with data from 7 mice in each control group (30 days and 60 days p.i.) and from 8 and 6 animals in the HTLV-1-infected groups of 30 days and 60 days p.i., respectively. Normal distribution of data was tested by Kolmogorov-Smirnov test and data were expressed as the mean ± standard deviation. Differences between groups were calculated by Student’s *t*-test. When equal variances were not assumed by Levene’s test, adjusted *p*-values were selected. Correlation between variables was determined by two-tailed Pearson correlation coefficient and linear regression (*R*^2^). Two-tailed *p*-value <0.05 was considered significant. 

## 3. Results

### 3.1. HTLV-1 PVL in the Blood, Spleen, and MLN of HTLV-1-Infected HIS-NSG Mice

**Figure 1:**
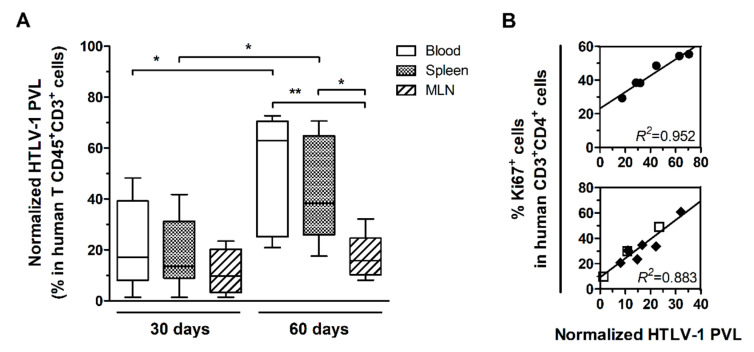
HTLV-1 proviral load in infected HIS-NSG mice. Blood, spleen, and mesenteric lymph nodes (MLN) from HTLV-1-infected mice were collected 30 days (*n* = 8) and 60 days (*n* = 6) post-infection (p.i.). (**A**) HTLV-1 proviral load (PVL) was determined by quantitative PCR in total DNA and then normalized by the frequency of human T-cells (CD45^+^CD3^+^ cells). Statistical analysis was performed with Student’s *t*-test (*, *p* < 0.05; **, *p* < 0.01). (**B**) Proliferating CD4^+^ T-cells were identified as Ki67^+^ cells by flow cytometry after intracellular staining of fresh cells. Linear regression (*R^2^*) between the normalized PVL and the frequency of Ki67^+^CD4^+^ T-cells is shown in the spleen (●) of mice at 60 days p.i. and in the MLN of mice at 30 days (□) and 60 days p.i. (◆) combined.

HIS-NSG mice were infected with HTLV-1 by inoculation with γ-irradiated MT2 cells or mock-infected by injection with PBS and euthanized after 30 days or 60 days of infection. The HTLV-1 PVL was measured in DNA samples from blood, spleen, and MLN and the values were normalized to the frequency of human T-cells (CD45^+^CD3^+^ cells). After 30 days, no significant difference was observed between samples from distinct sources (Figure 1A). At 60 days, the normalized PVL (nPVL) was significantly increased in blood and spleen compared to levels at 30 days. The PVL in these compartments was higher than in MLN (Figure 1A). No correlation was observed between the frequency of CD4^+^ T-cells and the nPVL (data not shown), but nPVL positively correlated with the frequency of CD4^+^ T-cells proliferating in the spleen after 60 days of infection (Linear regression, *R^2^* = 0.952, *p* = 0.001) as shown by Ki67 staining (Figure 1B). This result suggested that the expanding population of splenic CD4^+^ T-cells was predominantly infected. On the other hand, the nPVL did not increase in MLN (Figure 1A) but presented a strong positive correlation with the frequency of CD4^+^Ki67^+^ T-cells (Linear regression, *R^2^* = 0.883, *p* < 0.001) (Figure 1B).

After 60 days of infection, three of six HIS-NSG mice presented markedly high nPVL in the blood and spleen. In addition, high frequency of CD4 T-cells expressing CD25, a T-cell activation marker, was also observed (additional file: Table S1). These three mice also displayed splenomegaly, as evidenced by increased spleen weight (309.57 ± 76.59 mg) in comparison with mock-infected mice (77.80 ± 9.48 mg; Student’s *t*-test, *p* = 0.034) and mice after 30 days of infection (193.54 ± 37.88 mg; Student’s *t*-test, *p* = 0.007).

### 3.2. Expansion of CD4^+^ and CD8^+^ T-Cell Subsets Is Stimulated by HTLV-1 Infection

**Figure 2:**
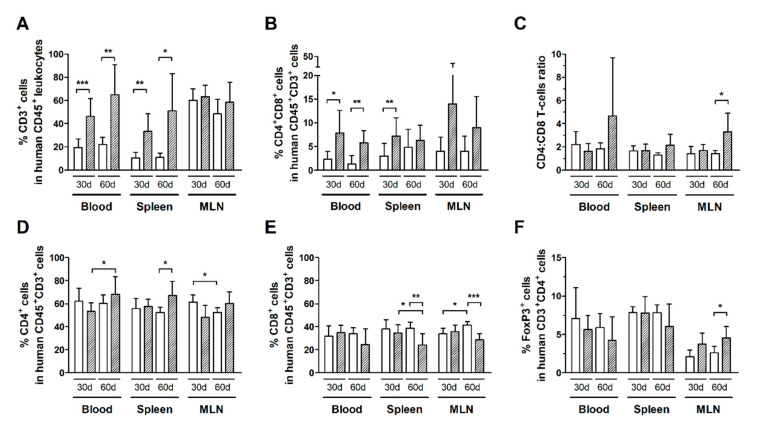
Frequency of T-cell subsets in HTLV-1-infected and normal HIS-NSG mice. Blood, spleen, and mesenteric lymph nodes (MLN) from control (white box) and HTLV-1-infected HIS-NSG mice (dashed box) were collected at 30 days and 60 days from inoculation. Human T-cell subsets were determined by flow cytometry according to the gating strategy shown in the additional file: Figure S1. (**A**) The frequency of T-cells (CD3^+^ cells) is shown in human leukocytes (CD45^+^ cells), and that of (**B**) CD4^+^CD8^+^ double-positive cells is shown in human T-cells (CD45^+^CD3^+^ cells). (**C**) The CD4:CD8 ratio represents the proportion between CD4^+^ and CD8^+^ single-positive T-cells, and (**D**) the frequencies of CD4^+^ and (**E**) CD8^+^ single-positive cells are shown in human CD45^+^CD3^+^ cells. (**F**) The frequency of FoxP3^+^ cells is shown in CD4^+^ T-cells. Results are shown as mean percentage with standard deviation bars. Statistical analysis was performed with Student’s *t*-test by pairwise comparisons, and adjusted *p*-values were selected when equal variances were not assumed by Levene’s test (*, *p* < 0.05; **, *p* < 0.01; ***, *p* < 0.001). Control groups 30 d and 60 d: *n* = 7; HTLV-1-infected group 30 d: *n* = 8; HTLV-1-infected group 60 d: *n* = 6.

Infected HIS-NSG mice had a higher frequency of total T-cells (CD3^+^ cells) compared to controls after 30 days of infection, as shown in the blood, spleen, but not in MLN (Figure 2A). In addition, infected mice displayed an increased subset of CD4^+^CD8^+^ double-positive T-cells in the blood and spleen (Figure 2B). This population was mainly constituted of CD8^high^CD4^low^ T-cells, which indicates activation of CD8^+^ T-cells [34] (Additional file: Figure S1b–d). Nonetheless, no change was observed in CD4:CD8 ratios, suggesting that both subsets were expanding proportionally (Figure 2C).

An elevated frequency of T-cells was detected in the blood and spleen, but not in MLN after 60 days of infection (Figure 2A). Indeed, the frequency of CD4^+^ T-cells in the blood of infected mice increased from day 30 to day 60 of infection (Figure 2D). The frequency of CD4^+^ T-cells was also increased in the spleen (Figure 2D), while that of CD8^+^ T-cells was reduced in comparison with controls (Figure 2E). Moreover, the frequency of CD8^+^ T-cells was also reduced in MLN (Figure 2E).

The frequency of distinct T-cell subsets in control HIS-NSG mice was steady, even after 60 days of mock-infection (Figure 2). On the other hand, infected mice had an increase in the CD4:CD8 ratio in MLN (Figure 2C), which was likely associated with the proliferation of CD4^+^ T-cells as shown by Ki67 expression (Figure 1B). Meanwhile, no change was observed in the frequency of CD4^+^FoxP3^+^ T-cells in the blood and spleen of HIS-NSG mice after 30 days or 60 days of infection, except an increase in MLN (Figure 2F). Although we have not performed functional assays, it is possible that the suppressive activity of these cells contributed to downregulation of the immune responses against infected cells, thus contributing to viral escape and proliferation of infected CD4^+^ T-cells.

### 3.3. HTLV-1-Infection Induces CD4^+^ and CD8^+^ T-Cells to Mature into Effector Memory Cells

**Figure 3:**
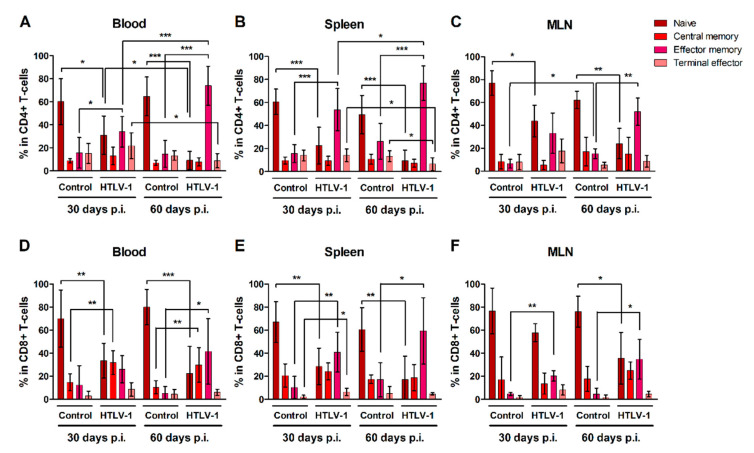
Maturation of CD4^+^ and CD8+ T-cells in HTLV-1-infected HIS-NSG mice. HTLV-1-infected and mock-infected (control) HIS-NSG mice were euthanized at 30 days and 60 days post-inoculation (p.i.). Blood, spleen, and mesenteric lymph nodes (MLN) were collected and cells were stained for flow cytometry analysis. (**A**–**C**) CD4^+^ and (**D**–**F**) CD8^+^ T-cell subsets were defined according to the gating strategy shown in the additional file: Figure S2: Naïve (CD45RA^+^CCR7^+^); central memory (CD45RA^−^CCR7^+^); effector memory (CD45RA^−^CCR7^−^); and terminal effector (CD45RA^+^CCR7^−^) cells. Results are shown as mean percentage with standard deviation bars. The statistical analysis was performed with Student’s *t*-test by pairwise comparisons, and adjusted *p*-values were selected when equal variances were not assumed by Levene’s test (*, *p* < 0.05; **, *p* < 0.01; ***, *p* < 0.001). Control groups 30 d and 60 d: *n* = 7; HTLV-1-infected group 30 d: *n* = 8; HTLV-1-infected group 60 d: *n* = 6.

CD4^+^ and CD8^+^ T-cells were characterized as naïve, central memory, effector memory and terminal effector cells according to surface expression of CD45RA and CCR7 (Additional file: Figure S2). Overall, HIS-NSG mice presented stable levels of both naïve and mature CD4^+^ (Figure 3A–C) and CD8^+^ T-cells (Figure 3D–F) over time, with a predominance of naïve T-cells. HTLV-1 infection triggered the differentiation of both naïve CD4^+^ and CD8^+^ T-cells, particularly into effector memory cells, as observed in the blood, spleen, and MLN after 30 and 60 days of infection when compared to controls. The frequency of effector memory CD4^+^ T-cells progressively increased in the blood (Figure 3A) and spleen (Figure 3B) of infected mice, but not in MLN (Figure 3C). By contrast, the population of terminal effector CD4^+^ T-cells in infected mice decreased in the blood (Figure 3A) and spleen (Figure 3B) between 30 and 60 days of infection, perhaps as a result of a shorter half-life or a change in cell maturation towards the effector memory phenotype.

Infection also prompted an expansion of central memory CD8^+^ T-cells, which reached a higher frequency in the blood (Figure 3D). After 30 days of infection, a small fraction of CD8^+^ T-cells was also stimulated to mature into terminal effector cells, as observed in the spleen (Figure 3E), although this process was not sustained up to 60 days.

### 3.4. Expression of Th1-Related Chemokine Receptors in T-Cells during HTLV-1 Infection

**Figure 4:**
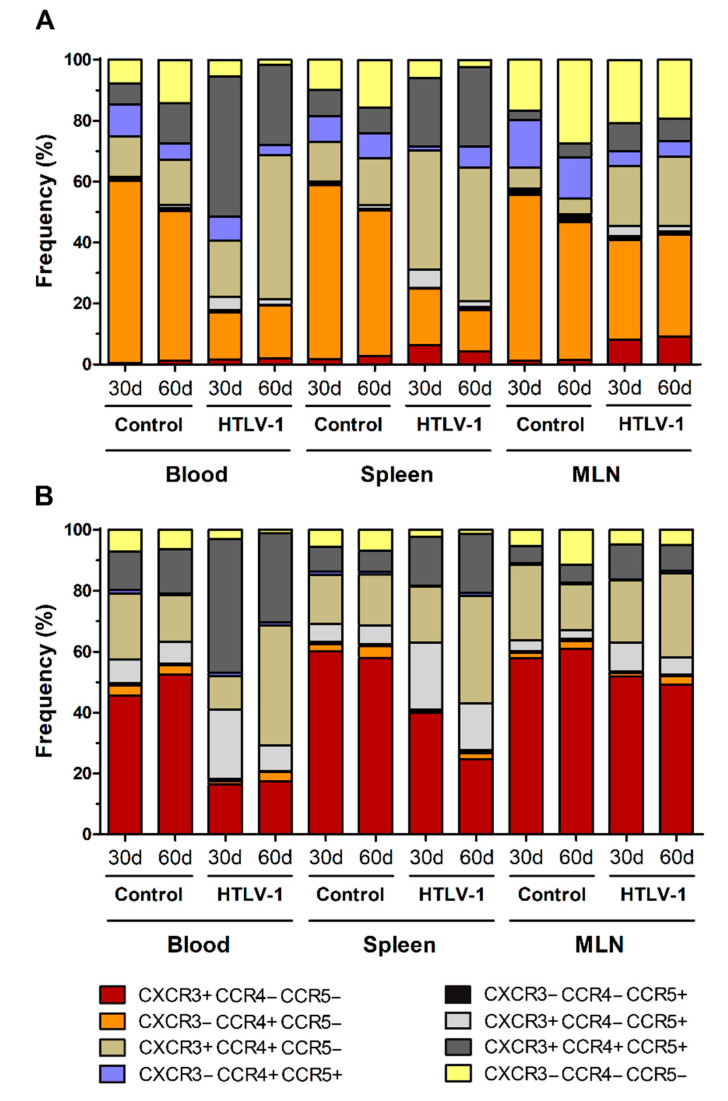
Expression of chemokine receptors in CD4^+^ and CD8^+^ T-cells. Blood, spleen, and mesenteric lymph nodes (MLN) from HTLV-1-infected and control HIS-NSG mice were collected at 30 days and 60 days from inoculation. Expression of CCR4, CCR5, and CXCR3 in (**A**) CD4^+^ and (**B**) CD8^+^ T-cells was evaluated by flow cytometry. Results are shown as mean frequency. Control groups 30 d and 60 d: *n* = 7; HTLV-1-infected group 30 d: *n* = 8; HTLV-1-infected group 60 d: *n* = 6.

HTLV-1 infection is associated with a strong Th1 response, and infected cells show altered expression of chemokine receptors, particularly CCR4 and CXCR3. In addition to CCR5, a receptor also related to Th1 responses, these chemokines receptors were evaluated in HIS-NSG to highlight the dynamics of their expression during early HTLV-1 infection. Most CD4^+^ T-cells in the blood, spleen, and MLN of control HIS-NSG mice expressed CC-chemokine receptor 4 (CCR4) without the presence of CCR5 and CXC-chemokine receptor 3 (CXCR3) (Figure 4A), which were indeed upregulated upon infection.

After 30 days, the subset of CXCR3^+^CCR5^+^CCR4^+^ CD4^+^ T-cells had significantly increased in the blood (Student’s *t*-test, *p* < 0.001) and spleen (Student’s *t*-test, *p* = 0.002) (Figure 4A and additional file Table S2). In fact, these cells became the predominant cell type in blood, while CXCR3^+^CCR4^+^ CD4^+^ T-cells formed the majority in the spleen (Student’s *t*-test, *p* < 0.001). In turn, the latter population was significantly elevated in the blood of mice after 60 days of infection compared with the day 30 levels (Student’s *t*-test, *p* = 0.038). Interestingly, this was followed by a decline of CCR5^+^ CD4^+^ T-cells. Virus infection also upregulated CXCR3 expression in CD4^+^ T-cells in MLN, as shown by the expansion of CXCR3^+^ (Student’s *t*-test, *p* = 0.002), CXCR3^+^CCR4^+^ (Student’s *t*-test, *p* = 0.007) and CXCR3^+^CCR5^+^CCR4^+^ CD4^+^ T-cell subsets (Student’s *t*-test, *p* = 0.001). Overall, this indicates an induction of expression of chemokine receptors related to a Th1 phenotype. However, a great proportion of CD4^+^ T-cells in MLN remained as single CCR4^+^ after 30 days (32.68 ± 1.11%) and 60 days of infection (33.44 ± 13.63%) (Additional file: Table S2).

CD8^+^ T-cells in the blood, spleen, and MLN of normal HIS-NSG mice were mostly characterized by CXCR3 expression in the absence of CCR4 and/or CCR5 (Figure 4B). However, infection induced the expression of CCR4 and CCR5 in these cells. After 30 days of infection, CXCR3^+^CCR5^+^CCR4^+^ CD8^+^ T-cells were predominant in the blood (Student’s *t*-test, *p* = 0.001), as reported for CD4^+^ T-cells (Figure 4A). An increase of CXCR3^+^CCR5^+^ CD8^+^ T-cells was also observed in the blood (Student’s *t*-test, *p* = 0.001), spleen (Student’s *t*-test, *p* = 0.002) and MLN (Student’s *t*-test, *p* = 0.016) (Figure 4B and additional file: Table S3). After 60 days of infection, CD8^+^ T-cells expressing CCR5 decreased in the blood and MLN, particularly the population of CXCR3^+^CCR5^+^ CD8^+^ T-cells (Student’s *t*-test: blood, *p* = 0.004; MLN, *p* = 0.045) (Figure 4B and additional file: Table S3). In blood, this was followed by a concomitant increase of the CXCR3^+^CCR4^+^ CD8^+^ T-cell subset (Student’s *t*-test, *p* < 0.001) (Figure 4B and additional file: Table S3), indicating a switch in the expression of chemokine receptors or the immigration of CD8^+^ T-cells.

### 3.5. HTLV-1 Infection Induces the Activation and Exhaustion of T-Cells in HIS-NSG Mice

**Figure 5:**
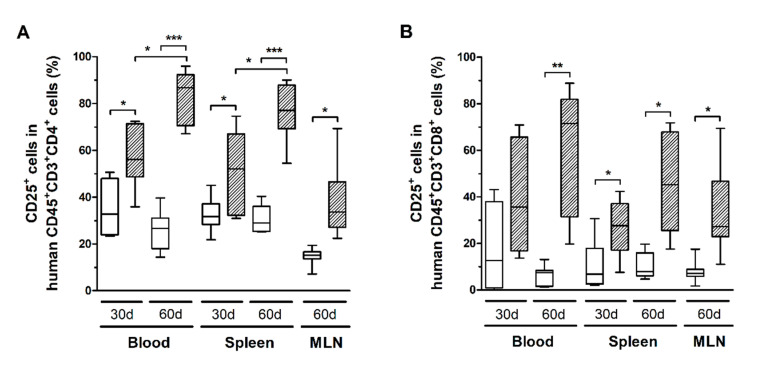
CD25 expression in T-cell subsets. Blood, spleen, and mesenteric lymph nodes (MLN) from control (white box) and HTLV-1-infected HIS-NSG mice (dashed box) were sampled at 30 days and 60 days from inoculation. CD25 expression in (**A**) CD4^+^ and (**B**) CD8^+^ human T-cells (CD45^+^CD3^+^ cells) was determined by flow cytometry. Statistical analysis was performed with Student’s *t*-test (*, *p* < 0.05; **, *p* < 0.01; ***, *p* < 0.001). Control groups 30 d and 60 d: *n* = 7; HTLV-1-infected group 30 d: *n* = 8; HTLV-1-infected group 60 d: *n* = 6.

HTLV-1 infection also triggered the activation of CD4^+^ and CD8^+^ T-cells. After 30 days, CD4^+^ T-cells in blood and spleen expressed significantly higher levels of CD25 compared to controls, which remained high up to 60 days of infection (Figure 5A). In fact, a direct comparison of the time points indicated that CD25 expression increased over time (Figure 5A). It is known that CD25 expression is induced in HTLV-1-infected cells. Although no correlation was observed between the nPVL and the frequency of CD4^+^CD25^+^ T-cells in blood, spleen, and MLN after 30 days of infection (data not shown), a positive correlation was observed with CD4^+^Ki67^+^ T-cells only in MLN (Pearson *R* = 0.995, *p* = 0.005). After 60 days of infection, the nPVL correlated with the frequency of CD4^+^CD25^+^ T-cells (Pearson *R* = 0.977, *p* = 0.001) only in blood but not with CD4^+^Ki67^+^ T-cells. In addition, these cells displayed an effector memory phenotype (Pearson *R* = 0.904, *p* = 0.012), suggesting that the population of infected cells was mainly constituted by CD4^+^ T-cells with an activated and/or mature phenotype.

**Figure 6:**
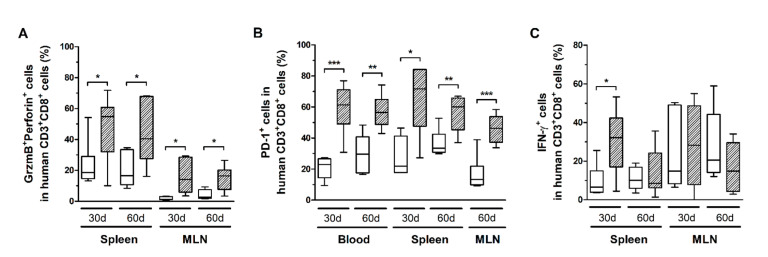
Granzyme B, perforin, IFN-γ, and PD-1 expression in CD8^+^ T-cells. Blood, spleen, and mesenteric lymph nodes (MLN) from control (white box) and HTLV-1-infected HIS-NSG mice (dashed box) were collected at 30 days and 60 days from inoculation. The frequency of CD8^+^ T-cells expressing (**A**) Granzyme B (GrzmB) and perforin, (**B**) PD-1, and (**C**) IFN-γ was determined by flow cytometry. The surface expression of PD-1 was detected in non-stimulated cells, while GrzmB, perforin, and IFN-γ were intracellularly stained after 6 h of stimulation with phorbol-myristate-acetate and ionomycin. Frequency is shown within CD8^+^ T-cells. Statistical analysis was performed with Student’s *t*-test (*, *p* < 0.05; **, *p* < 0.01; ***, *p* < 0.001). Control groups 30 d and 60 d: *n* = 7; HTLV-1-infected group 30 d: *n* = 8; HTLV-1-infected group 60 d: *n* = 6.

CD8^+^ T-cells in infected mice also exhibited increased CD25 expression (Figure 5B), as well as higher levels of granzyme B and perforin (Figure 6A). However, no correlation with the PVL was observed in the blood, spleen, and MLN at any time point (data not shown). Nonetheless, infected mice presented CD8^+^ T-cells with increased and persistently higher rates of PD-1 expression when compared to controls (Figure 6B). Moreover, IFN-γ expression was initially elevated in CD8^+^ T-cells from the spleen of infected mice, but it returned to levels similar to those of mock-infected controls after 60 days (Figure 6C). Therefore, it is possible that downregulation triggered by PD-1 signaling have impaired the function of CD8^+^ T-cells. To corroborate this, we analyzed the frequency of mDCs and the expression of PD-1 ligand (PD-L1) and co-stimulatory molecules CD80 and CD86.

**Figure 7:**
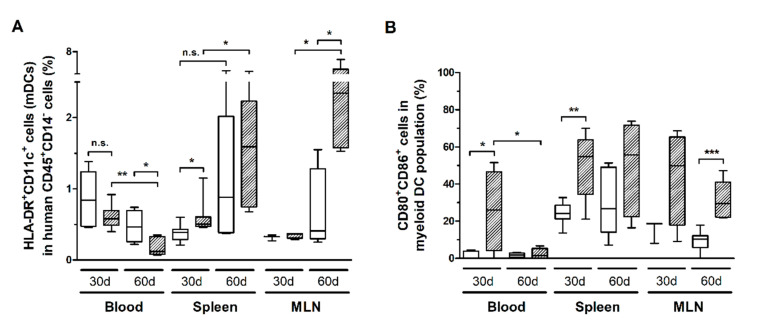
Frequency of myeloid dendritic cells and the expression of co-stimulatory molecules. Blood, spleen, and mesenteric lymph nodes (MLN) from HTLV-1-infected (dashed box) and control HIS-NSG mice (white box) were collected at 30 days and 60 days from inoculation. (**A**) The frequency of myeloid dendritic cells (mDCs), which were identified as HLA-DR^+^CD11c^+^ cells, was determined in the gate of CD45^+^CD14^−^ cells. (**B**) Frequency of CD80^+^CD86^+^ cells is shown within mDCs. Statistical analysis was performed with Student’s *t*-test (n.s., non-significant; *, *p* < 0.05; **, *p* < 0.01; ***, *p* < 0.001). Control groups 30 d and 60 d: *n* = 7; HTLV-1-infected group 30 d: *n* = 8; HTLV-1-infected group 60 d: *n* = 6.

HIS-NSG mice infected with HTLV-1 presented a progressive reduction of HLA-DR^+^CD11c^+^ mDCs in the blood (Figure 7A). However, it was not possible to determine whether mDCs were depleted by infection or that they had migrated to the tissues. The latter scenario is supported by the finding that the reduced mDC frequency in blood was followed by a concomitant increase in the spleen and MLN (Figure 7A). In addition, these cells displayed elevated expression of CD80 and CD86 in the blood and spleen after 30 days of infection, which remained high in the spleen, and particularly in the MLN, after 60 days (Figure 7B). Therefore, we assume that activated mDCs migrated to the periphery. A significant fraction of mDCs expressed PD-L1, although no change over time was observed (additional file: Figure S3a). Nevertheless, blood mDCs of infected mice showed increased PD-L1 expression after 30 days of infection, as shown by the MFI, which returned to control levels after 60 days (additional file: Figure S3b).

## 4. Discussion

The development of murine models that support HTLV-1 infection has allowed the study of events related to the establishment of ATLL [25,26,27,28,29,30,35]. The development of ATLL is observed in 2–7% of HTLV-1 carriers following a latency period of approximately 20–30 years, which involves several oncogenic steps including viral and cellular epigenetic changes [21,36,37]. Therefore, animal models are an important tool not only to understand the initiation and progression of events underlying the in vivo leukemogenic process, but also to carry out preclinical studies with candidate therapeutic agents. However, limitations in animal models have hampered the detailed analysis of the complex interactions between the virus and the host immune responses as observed in humans.

Murine cells are not permissive to HTLV-1 [26] and productive infection in humans mainly involves T-cells, particularly CD4^+^ T-cells. The HIS-NSG mouse model supported the HTLV-1 infection, and a strong proliferation of CD4^+^ T-cells was observed after 60 days from inoculation. A significant proportion of ATLL patients displays lymphadenopathy and 50% have hepatosplenomegaly [38]. In agreement with characteristics observed in humans, high HTLV-1 PVL, splenomegaly, and high CD25 expression in CD4^+^ T-cells were observed in this mouse model. Moreover, the proliferation of CD4^+^ T-cells initially correlated with the PVL in MLN as well as later in the spleen, indicating that lymph nodes may represent a site of expansion of infected CD4^+^ T-cells. However, a limitation of this study is the impossibility to identify whether proliferating cells represented infected cells or antigen-stimulated cells by immune responses triggered by the infection.

Chronic T-cell activation with high CD25 expression is a hallmark of HTLV-1 infection and the frequency of CD4^+^CD25^+^ T-cells correlates positively with the PVL in the peripheral blood of HTLV-1 carriers [9,39]. However, this association was not observed in HIS-NSG mice after 30 days of infection (data not shown). CD25 expression was likely upregulated in T-cells by concomitant events associated with cellular infection and immune activation. This was corroborated by migration of activated CD80^+^CD86^+^ mDCs to the spleen and MLN of the HTLV-1-infected mice. However, humanized NSG mice have a reduced development of monocytes/macrophages and are virtually absent of granulocytes [40,41]. In addition, CD14^+^ monocytes from neonatal humanized NSG mice show a reduced capacity for T cell co-stimulation but without losing the ability for phagocytosis or cytokine secretion [40]. de Castro-Amarante et al. [42] also showed that monocytes are involved in the viral spread in HTLV-1 infection. Therefore, such impairments limited our analysis in the context of interactions between monocytes/macrophages and T-cells.

On the other hand, at 60 days, HTLV-1 PVL showed a significant positive correlation with the frequency of CD4^+^ T-cells expressing CD25 and Ki67, indicating the proliferation of infected cells and the establishment of chronic infection. In turn, early activation of T-cells was also indicated by elevated levels of double-positive T-cells, predominantly CD4lowCD8high, in the blood, spleen, and MLN of infected mice. Pérès et al. [29] also observed a significant increase of double-positive T-cells in humanized mice infected with HTLV-1 after 7 weeks. CD4^+^CD8^+^ T-cells are present in small numbers in the peripheral blood of healthy humans [43]. In general, these cells are functional antigen-specific effector/memory CD4^+^ or CD8^+^ T-cells re-expressing CD8 or CD4, respectively [44]. Indeed, there is considerable evidence of their increase during chronic viral infections. Expansion of double-positive T-cells has been described in individuals infected by HIV [45], HBV, and HCV [46]. An increased frequency of CD4highCD8low T-cells was observed in HCV infection, while HBV-infected individuals displayed higher levels of CD4lowCD8high and CD4highCD8high double-positive T-cells [46]. Macchi et al. [47] reported the emergence of double-positive T-cells as an early event following in vitro infection of human PBMCs by HTLV-1, particularly the re-activation of CD4 expression by CD8^+^ T-cells, which corroborates our in vivo findings.

Villaudy et al. [26] demonstrated that HTLV-1 infection accelerates the thymic development of T-cells in humanized BRG mice, especially of CD4^+^ T-cells. Here, we showed that infection of HIS-NSG mice strongly induced the differentiation of naïve CD4^+^ and CD8^+^ T-cells into effector memory cells. The differentiation status of a T-cell influences its surviving potential, which is determined by the rate of proliferation and the susceptibility to apoptosis. During differentiation, T-cells display distinct phenotypes and functions as they progress in a linear flow along subtypes: naïve > stem central memory > central memory > transitional memory > effector memory > terminal effector cells [48]. In humans, HTLV-1 infects CD4^+^ and CD8^+^ T-cells, and CD45RO^+^ memory cells represent the main reservoir of infected cells [6]. In the peripheral blood of healthy individuals, T-cells are largely quiescent as evaluated by Ki67 expression [49], a nuclear antigen expressed by proliferating cells irrespective of the cell cycle phase [50]. In turn, most Ki67^+^ T-cells harbor an effector memory phenotype ex vivo [49,51]. In addition, these cells undergo faster turnover compared to naïve T-cells as demonstrated by in vivo isotope labeling with deuterated glucose [52]. In HIS-NSG mice, HTLV-1 PVL positively correlated with the frequency of Ki67^+^CD4^+^ T-cells in the spleen and MLN after 60 days of infection. We also observed a positive correlation between the PVL and the frequency of effector memory CD4^+^ T-cells. Taken together, these results indicate that the population of HTLV-1-infected cells was mainly constituted by effector memory CD4^+^ T-cells undergoing rapid expansion. However, it was not possible to determine whether cells were prompted to differentiate because of a direct effect of infection or due to mechanisms associated with the development of an antiviral immune response.

ATLL cells express FoxP3 in 50% to 80% of cases [53,54], which is a transcription factor associated with CD4^+^ Treg cells [55]. However, ATLL cells do not exhibit immunosuppressive functions [56]. In fact, these cells present low FoxP3 levels, whose expression was shown to be induced by the viral HBZ protein [24]. Moreover, HAM/TSP patients and HTLV-1 asymptomatic carriers have an increased frequency of functional CD4^+^FoxP3^+^ Treg cells, which can suppress virus-specific responses mediated by CD8^+^ T-cells [57]. This feature was not apparent in infected HIS-NSG mice. By contrast, persistent exposure to viral antigens induced a strong activation of CD8^+^ T-cells, which was evident by the high frequency of cells expressing PD-1, CD25, perforin, and granzyme B. PD-1 expression is upregulated upon cell activation, particularly after the effector T-cell stage, and in CD8^+^ T-cells this is involved in the process of cell exhaustion [58]. Indeed, PD-1 signaling downregulates the cytotoxic activity of virus-specific CD8^+^ T-cells and it has a central role in T-cell dysfunction in chronic infections, particularly by HIV [59,60], HBV [61] and HCV [62,63]. This pathway is triggered by interaction with PD-L1, which is widely expressed on both hematopoietic and parenchymal cells [58]. Coincidently, increased PD-L1 expression on mDCs after 30 days of infection was followed by a decrease in splenic IFN-γ^+^CD8^+^ T-cells. Therefore, it is possible that CD8^+^ T-cells in infected mice acquired an exhausted state, which impaired viral clearance and favored the establishment of chronic infection. However, further investigation of other molecules associated with T-cell exhaustion, such as LAG-3, CD244 (2B4), CD160, and TIM-3 should reveal the exact level of dysfunction.

The ability of T-cells to migrate from the periphery into the tissues is governed by expression of distinct combinations of adhesion molecules and chemokine receptors, which is defined during initial antigen priming [64]. HTLV-1-infected individuals display a strong Th1 response [5], and infected HIS-NSG mice presented an enhanced expression of CXCR3 and CCR5. Chemokine receptors are distinctly expressed according to the T-cell phenotype. In homeostatic conditions, CCR4 is expressed by Th2, Treg, and Th17 cells and directs migration towards the skin or sites with increased levels of CCL17 and CCL22 [65]. However, despite the Th1 phenotype and IFN-γ expression, infected CD4^+^ T-cells have been characterized as CCR4^+^ in patients with HAM/TSP [66] and ATLL [67]. Indeed, it was shown that CD4^+^CCR4^+^ T-cells represent the main reservoir of HTLV-1-infected cells [66]. Sugata et al. [68] showed that CCR4 expression on ATLL cells is stimulated by HBZ protein through induced GATA3 expression. In addition, Tax protein was shown to upregulate CCL22 expression, thus enhancing the cell-to-cell transmission of HTLV-1 to CCR4^+^CD4^+^ T-cells by selectively attracting them [69]. As most CD4^+^ T-cells in HIS-NSG mice are CCR4^+^, this feature may have made these mice highly permissive to HTLV-1 infection.

## 5. Conclusions

HIS-NSG mice supported HTLV-1 infection, with a strong expansion of activated T-cells in the blood and lymphoid tissues. Activation of CD8^+^ T-cells was followed by expression of granzyme B and perforin, and IFN-γ response in these cells was limited probably due to downregulation associated with engagement of the PD-1/PD-L1 pathway. Many aspects of chronic HTLV-1 infection in humans were observed in this model, such as splenomegaly, proliferation of effector/memory CD4^+^ and CD8^+^ T-cells and polarization of the immune response to T-cell phenotypes associated with the expression of CXCR3 and CCR5. However, these characteristics are also associated with the development of cellular immune responses. Our findings on the modulation of T-cells in early stages of infection showed that several phenotypic characteristics of these cells upon immune activation are shared with HTLV-1-infected T-cells. Thus, we suggest that HTLV-1 modulates the host immune responses to favor the establishment of viral persistence. This HIS-NSG model seems very useful for investigating events associated with HTLV-1 infection and for conducting preclinical therapy studies.

## Data Availability

The data presented in this study are available upon request from the corresponding author.

## Supplementary Materials

The following are available online at https://www.mdpi.com/article/10.3390/cells10102514/s1, Figure S1: Frequency of T-cell subsets, Figure S2: Maturation of CD4+ and CD8+ T-cells, Figure S3: Expression of PD-L1 in myeloid dendritic cells (mDCs), Table S1: Lymphoproliferation in HTLV-1-infected HIS-NSG mice, Table S2: Expression of chemokine receptors in CD4+ T-cells, Table S3: Expression of chemokine receptors in CD8+ T-cells.
