# Supplementary material for: Early Effects of HTLV-1 Infection on the Activation, Exhaustion, and Differentiation of T-Cells in Humanized NSG Mice
Source: Cells. 2021 Sep 23;10(10):2514. doi: 10.3390/cells10102514 (PMC8534134; doi:10.3390/cells10102514)
Supplement: Supplementary file 1 [file cells-10-02514-s001.zip › cells-1324802-supplementary.pdf]

Table S1. Lymphoproliferation in HTLV-1-infected HIS-NSG mice.

Mouse ID	Normalized HTLV-1 PVL (% HTLV-1$^+$ cells in CD3$^+$ cells)		CD3$^+$ cells (%)		CD4$^+$CD25$^+$ cells (%)		Spleen weight (mg)
	Blood	Spleen	Blood	Spleen	Blood	Spleen	
**30 days p.i.**							
B1_F_LK	48.19	41.79	44.60	29.60	58.98	60.08	211.0
B1_F_LT	1.38	1.36	40.70	19.30	53.34	45.86	197.4
B1_F_NO	15.98	8.40	44.30	26.90	52.95	58.31	196.8
B1_F_RT	18.17	34.55	36.30	15.40	35.87	31.33	125.0
D1_F_RK	7.52	21.30	76.60	56.80	71.06	69.30	203.3
D1_F_TT	9.58	15.00	61.60	44.50	72.43	74.58	204.2
D1_F_LK	25.69	12.10	31.00	50.00	-	30.85	252.9
D1_F_RT	43.72	10.26	33.70	23.20	-	35.01	157.7
**60 days p.i.**							
B2_F_TT	20.89	31.88	45.80	23.90	67.07	54.5	121.3
D2_F_NO*	63.77	44.68	77.30	65.00	89.92	78.59	378.8
D2_F_TT*	72.69	70.60	96.40	90.30	95.94	90.00	322.6
D2_M_NO*	69.81	62.96	88.50	82.90	91.04	87.14	227.3
D2_F_RT	62.06	28.69	48.70	23.10	83.64	75.61	65.1
D2_F_LT	26.51	17.56	33.30	19.90	71.72	74.14	104.0

HIS-NSG mice were infected with HTLV-1. After 30 days and 60 days post-infection, blood and spleen were collected. HTLV-1 proviral load (PVL) was determined by quantitative PCR in total DNA and then normalized by the frequency of human T-cells (CD3$^+$ cells). The frequency of T-cells was determined within human leukocytes (CD45$^+$ cells), and the frequency of CD25$^+$ cells is shown within CD4$^+$ T-cells, as defined by flow cytometry. Spleen weight is shown in milligrams (mg). *Mice with splenomegaly and augmented lymphoproliferation.

Table S2. Expression of chemokine receptors in CD4+ T-cells.

Time	Sample	Subsets	Groups		*p*-value[a]	*p*-value[b]	*p*-value[c]
			Control	HTLV-1			
30 days p.i.	Blood	CXCR3–CCR4–CCR5–	7.73 ± 2.91	5.36 ± 6.04	0.363	-	-
		CXCR3–CCR4+CCR5–	59.74 ± 9.91	15.46 ± 12.20	**<0.001**	-	-
		CXCR3+CCR4–CCR5–	0.43 ± 0.29	1.62 ± 1.30	**0.036**	-	-
		CXCR3–CCR4–CCR5+	0.91 ± 0.69	0.73 ± 1.17	0.722	-	-
		CXCR3+CCR4–CCR5+	0.45 ± 0.50	4.31 ± 2.27	**0.002**	-	-
		CXCR3+CCR4+CCR5–	13.29 ±6.90	18.60 ± 8.17	0.200	-	-
		CXCR3–CCR4+CCR5+	10.55 ± 2.27	7.72 ± 9.06	0.419	-	-
		CXCR3+CCR4+CCR5+	6.93 ± 2.89	46.20 ± 18.29	**<0.001**	-	-
	Spleen	CXCR3–CCR4–CCR5–	9.94 ± 9.56	5.86 ± 4.96	0.094	-	-
		CXCR3–CCR4+CCR5–	57.17 ± 10.32	18.51 ± 14.04	**<0.001**	-	-
		CXCR3+CCR4–CCR5–	1.69 ± 1.00	6.37 ± 2.32	**<0.001**	-	-
		CXCR3–CCR4–CCR5+	0.54 ± 0.30	0.23 ± 0.13	**0.037**	-	-
		CXCR3+CCR4–CCR5+	0.74 ± 0.52	5.99 ± 2.76	**0.001**	-	-
		CXCR3+CCR4+CCR5–	12.92 ± 6.00	39.14 ± 11.52	**<0.001**	-	-
		CXCR3–CCR4+CCR5+	8.45 ± 5.05	1.35 ± 1.07	**0.009**	-	-
		CXCR3+CCR4+CCR5+	8.56 ± 3.75	22.55 ± 8.32	**0.002**	-	-
	MLN	CXCR3–CCR4–CCR5–	16.73 ± 3.58	20.90 ± 6.28	0.356	-	-
		CXCR3–CCR4+CCR5–	54.49 ± 9.60	32.68 1.11	**0.006**	-	-
		CXCR3+CCR4–CCR5–	1.21 ± 0.92	8.09 ± 1.83	**0.002**	-	-
		CXCR3–CCR4–CCR5+	1.60 ± 1.51	1.38 ± 1.04	0.831	-	-
		CXCR3+CCR4–CCR5+	0.36 ± 0.15	3.35 ± 1.69	**0.031**	-	-
		CXCR3+CCR4+CCR5–	6.97 ± 3.19	19.67 ± 4.11	**0.007**	-	-
		CXCR3–CCR4+CCR5+	15.64 ± 13.27	4.90 ± 4.29	0.180	-	-
		CXCR3+CCR4+CCR5+	3.00 ± 0.40	9.03 ± 1.53	**0.001**	-	-
60 days p.i.	Blood	CXCR3–CCR4–CCR5–	14.27 ± 6.25	1.65 ± 2.54	**0.001**	**0.027**	0.186
		CXCR3–CCR4+CCR5–	49.04 ± 13.55	17.38 ± 13.49	**0.001**	0.118	0.785
		CXCR3+CCR4–CCR5–	1.32 ± 0.54	1.92 ± 1.72	0.446	**0.002**	0.718
		CXCR3–CCR4–CCR5+	0.88 ± 0.34	0.20 ± 0.17	**0.001**	0.913	0.299
		CXCR3+CCR4–CCR5+	1.14 ± 1.16	1.89 ± 1.31	0.301	0.174	**0.038**
		CXCR3+CCR4+CCR5–	14.78 ± 6.25	47.23 ± 14.71	**<0.001**	0.678	**0.001**
		CXCR3–CCR4+CCR5+	5.34 ± 1.79	3.37 ± 3.05	0.176	**<0.001**	0.285
		CXCR3+CCR4+CCR5+	13.22 ± 9.96	26.39 ± 16.57	0.104	0.134	0.059
	Spleen	CXCR3–CCR4–CCR5–	15.63 ± 3.65	2.42 ± 3.47	**<0.001**	**0.016**	0.173
		CXCR3–CCR4+CCR5–	47.66 ± 12.11	13.685 ± 16.84	**0.002**	0.154	0.569
		CXCR3+CCR4–CCR5–	2.79 ± 2.78	4.21 ± 3.04	0.417	0.348	0.157
		CXCR3–CCR4–CCR5+	0.57 ± 0.21	0.99 ± 1.77	0.576	0.827	0.341
		CXCR3+CCR4–CCR5+	1.21 ± 1.04	1.90 ± 1.52	0.383	0.317	**0.007**
		CXCR3+CCR4+CCR5–	15.52 ± 8.93	43.82 ± 25.80	**0.029**	0.544	0.654
		CXCR3–CCR4+CCR5+	8.18 ± 5.19	6.92 ± 10.17	0.793	0.925	0.238
		CXCR3+CCR4+CCR5+	8.44 ± 3.33	26.07 ± 22.55	0.114	0.953	0.689
	MLN	CXCR3–CCR4–CCR5–	27.56 ± 7.06	19.37 ± 6.54	0.054	**0.014**	0.722
		CXCR3–CCR4+CCR5–	45.11 ± 8.27	33.44 ± 13.63	0.084	0.154	0.915
		CXCR3+CCR4–CCR5–	1.52 ± 0.79	9.07 ± 7.83	0.065	0.604	0.816
		CXCR3–CCR4–CCR5+	2.03 ± 1.32	1.14 ± 0.84	0.183	0.658	0.692
		CXCR3+CCR4–CCR5+	0.53 ± 0.42	1.80 ± 0.85	**0.005**	0.525	0.087
		CXCR3+CCR4+CCR5–	5.30 ± 2.86	22.76 ± 5.92	**<0.001**	0.434	0.393
		CXCR3–CCR4+CCR5+	13.38 ± 1.84	5.17 ± 2.47	**<0.001**	0.796	0.914
		CXCR3+CCR4+CCR5+	4.57 ± 2.72	7.26 ± 3.15	0.126	0.182	0.334

HTLV-1-infected and control HIS-NSG mice were euthanized at 30 days and 60 days post-inoculation (p.i.). Blood, spleen and mesenteric lymph nodes (MLN) were collected and the expression of CXCR3, CCR4 and CCR5 chemokine receptors was evaluated in CD4+ human T- (CD45+CD3+ mononuclear cells) by flow cytometry. Results are shown as the mean percentage ± standard deviation. Statistical analysis was performed with Student's *t*-test by pairwise comparisons, and an adjusted *p*-value was selected when equal variances were not assumed by Levene's test. *P*-value<0.05 was considered significant (in bold). [a] Control *versus* HTLV-1 infection. [b] 30 days p.i. (n=7) *versus* 60 days p.i. (n=7) groups of control HIS-NSG mice. [c] 30 days p.i. (n=8) *versus* 60 days p.i. (n=6) groups of HTLV-1-infected HIS-NSG mice.

Table S3. Expression of chemokine receptors in CD8$^+$ T-cells.

Time	Sample	Subsets	Groups		*p*-value[a]	*p*-value[b]	*p*-value[c]
			Control	HTLV-1			
30 days p.i.	Blood	CXCR3–CCR4–CCR5–	7.13 ± 4.83	2.96 ± 2.53	0.071	-	-
		CXCR3–CCR4+CCR5–	3.41 ± 1.53	0.94 ± 1.05	**0.001**	-	-
		CXCR3+CCR4–CCR5–	45.54 ± 14.87	16.42 ± 10.99	**0.003**	-	-
		CXCR3–CCR4–CCR5+	0.80 ± 0.94	0.88 ± 1.15	0.885	-	-
		CXCR3+CCR4–CCR5+	7.74 ± 3.22	22.71 ± 9.31	**0.001**	-	-
		CXCR3+CCR4+CCR5–	21.70 ± 14.55	10.92 ± 2.71	0.099	-	-
		CXCR3–CCR4+CCR5+	1.02 ± 1.36	1.26 ± 1.70	0.770	-	-
		CXCR3+CCR4+CCR5+	12.67 ± 7.22	43.93 ± 18.88	**0.001**	-	-
	Spleen	CXCR3–CCR4–CCR5–	5.54 ± 3.94	2.39 ± 1.88	0.088	-	-
		CXCR3–CCR4+CCR5–	2.31 ± 0.74	0.79 ± 0.40	**<0.001**	-	-
		CXCR3+CCR4–CCR5–	60.06 ± 11.54	39.94 ± 13.70	**0.009**	-	-
		CXCR3–CCR4–CCR5+	0.76 ± 0.68	0.36 ± 0.16	0.128	-	-
		CXCR3+CCR4–CCR5+	5.92 ± 2.84	21.92 ± 9.99	**0.002**	-	-
		CXCR3+CCR4+CCR5–	16.20 ± 8.84	18.41 ± 8.24	0.625	-	-
		CXCR3–CCR4+CCR5+	1.05 ± 0.93	0.20 ± 0.09	0.052	-	-
		CXCR3+CCR4+CCR5+	8.16 ± 4.02	16.00 ± 8.81	**0.050**	-	-
	MLN	CXCR3–CCR4–CCR5–	5.27 ± 6.27	4.75 ± 1.47	0.901	-	-
		CXCR3–CCR4+CCR5–	1.83 ± 2.00	1.23 ± 0.77	0.594	-	-
		CXCR3+CCR4–CCR5–	57.78 ± 18.69	51.82 ± 13.35	0.640	-	-
		CXCR3–CCR4–CCR5+	0.57 ± 0.66	0.39 ± 0.28	0.637	-	-
		CXCR3+CCR4–CCR5+	3.43 ± 1.27	9.56 ± 2.73	**0.016**	-	-
		CXCR3+CCR4+CCR5–	24.93 ± 13.04	20.40 ± 10.85	0.635	-	-
		CXCR3–CCR4+CCR5+	0.57 ± 0.51	0.26 ± 0.25	0.339	-	-
		CXCR3+CCR4+CCR5+	5.65 ± 1.13	11.59 ± 4.33	0.066	-	-
60 days p.i.	Blood	CXCR3–CCR4–CCR5–	6.35 ± 2.59	1.10 ± 1.26	**0.001**	0.716	0.126
		CXCR3–CCR4+CCR5–	3.23 ± 2.44	3.11 ± 3.14	0.940	0.870	0.089
		CXCR3+CCR4–CCR5–	52.37 ± 18.81	17.37 ± 14.44	**0.003**	0.466	0.891
		CXCR3–CCR4–CCR5+	0.46 ± 0.26	0.34 ± 0.37	0.525	0.369	0.294
		CXCR3+CCR4–CCR5+	7.18 ± 2.72	8.30 ± 3.72	0.543	0.731	**0.004**
		CXCR3+CCR4+CCR5–	15.20 ± 4.58	39.57 ± 16.22	**0.013**	0.282	**<0.001**
		CXCR3–CCR4+CCR5+	0.72 ± 0.81	0.97 ± 1.23	0.671	0.626	0.731
		CXCR3+CCR4+CCR5+	14.49 ± 13.92	29.26 ± 12.89	0.074	0.765	0.129
	Spleen	CXCR3–CCR4–CCR5–	6.92 ± 2.79	1.38 ± 0.98	**0.003**	0.491	0.259
		CXCR3–CCR4+CCR5–	3.80 ± 3.15	1.84 ± 1.46	0.195	0.304	0.141
		CXCR3+CCR4–CCR5–	57.99 ± 10.24	24.71 ± 16.04	**0.002**	0.741	0.079
		CXCR3–CCR4–CCR5+	0.74 ± 0.25	1.08 ± 1.29	0.534	0.941	0.228
		CXCR3+CCR4–CCR5+	5.92 ± 1.97	15.42 ± 16.74	0.197	0.996	0.381
		CXCR3+CCR4+CCR5–	16.84 ± 5.19	35.14 ± 21.81	0.073	0.879	0.068
		CXCR3–CCR4+CCR5+	0.90 ± 0.40	1.06 ± 1.21	0.763	0.729	0.142
		CXCR3+CCR4+CCR5+	6.91 ± 2.60	19.37 ± 13.96	0.057	0.528	0.618
	MLN	CXCR3–CCR4–CCR5–	11.40 ± 4.21	4.93 ± 2.55	**0.007**	0.102	0.901
		CXCR3–CCR4+CCR5–	2.57 ± 0.71	2.74 ± 1.42	0.787	0.591	0.091
		CXCR3+CCR4–CCR5–	60.94 ± 10.45	49.22 ± 11.43	0.080	0.734	0.749
		CXCR3–CCR4–CCR5+	0.61 ± 0.70	0.50 ± 0.40	0.741	0.928	0.642
		CXCR3+CCR4–CCR5+	2.83 ± 2.17	5.61 ± 2.48	0.054	0.673	**0.045**
		CXCR3+CCR4+CCR5–	15.20 ± 7.73	27.57 ± 9.49	**0.025**	0.170	0.300
		CXCR3–CCR4+CCR5+	0.60 ± 0.64	0.93 ± 1.13	0.524	0.939	0.287
		CXCR3+CCR4+CCR5+	5.84 ± 3.71	8.51 ± 4.92	0.289	0.934	0.341

HTLV-1-infected and control HIS-NSG mice were euthanized at 30 days and 60 days post-inoculation (p.i.). Blood, spleen and mesenteric lymph nodes (MLN) were collected and the expression of CXCR3, CCR4 and CCR5 chemokine receptors was evaluated in CD8$^+$ human T- (CD45$^+$CD3$^+$ mononuclear cells) by flow cytometry. Results are shown as the mean percentage ± standard deviation. Statistical analysis was performed with Student's *t*-test by pairwise comparisons, and an adjusted *p*-value was selected when equal variances were not assumed by Levene's test. *P*-value<0.05 was considered significant (in bold).

[a] Control *versus* HTLV-1 infection. [b] 30 days p.i. (n=7) *versus* 60 days p.i. (n=7) groups of control HIS-NSG mice. [c] 30 days p.i. (n=8) *versus* 60 days p.i.

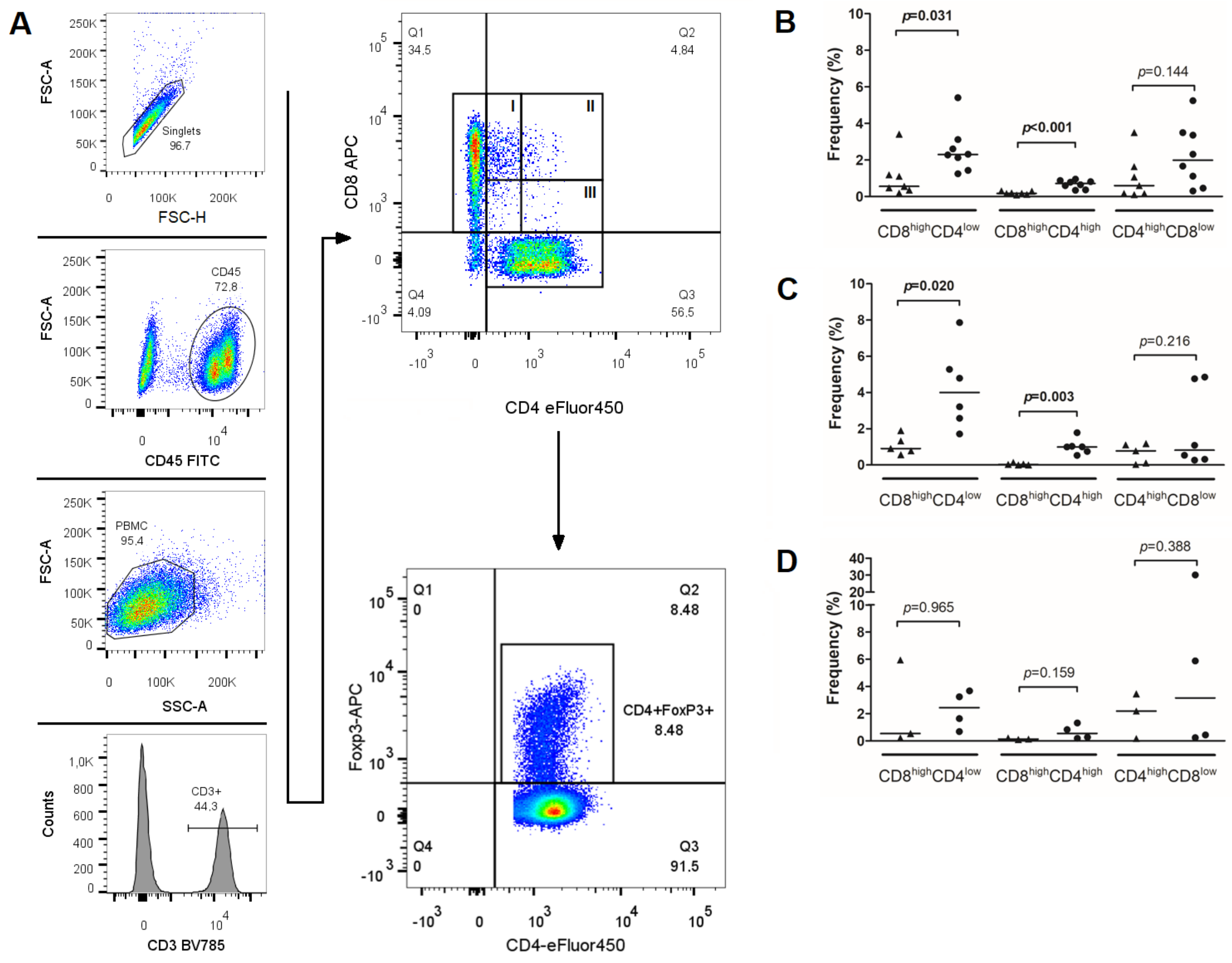

Figure S1. Frequency of T-cell subsets. HTLV-1-infected and mock-infected HIS-NSG mice were euthanized at 30 days and 60 days from inoculation, when blood, spleen and mesenteric lymph nodes (MLN) were collected. **(A)** Human leukocytes were stained with FITC-labeled anti-human CD45 monoclonal antibody and sorted by flow cytometry. After gating on CD45$^+$ cells, mononuclear cells were selected according to forward (FSC) and side scatter (SSC) properties. The frequency of T-cells (CD3$^+$ cells) was determined within CD45$^+$ mononuclear cells, while that of CD4$^+$, CD8$^+$ and CD4$^+$CD8$^+$ cells were defined within the gate of human T-cells (CD45$^+$CD3$^+$). The frequency of FoxP3$^+$ cells was determined within CD4$^+$ T-cells. CD4$^+$CD8$^+$ double-positive T-cells were further divided into: (I) CD8^{high}CD4^{low}, (II) CD8^{high}CD4^{high}, and (III) CD4^{high}CD8^{low} according to the intensity of the fluorescence signal. The frequency of these subsets is shown as the percentage of T-cells in the **(B)** blood, **(C)** spleen and **(D)** MLN of mock-infected (▲) and HTLV-1-infected (●) HIS-NSG mice at 30 days from inoculation. Statistical analysis was performed with Student's *t*-test, and adjusted *p*-value was chosen when variances were not equal. *P*-value<0.05 was considered significant (in bold).

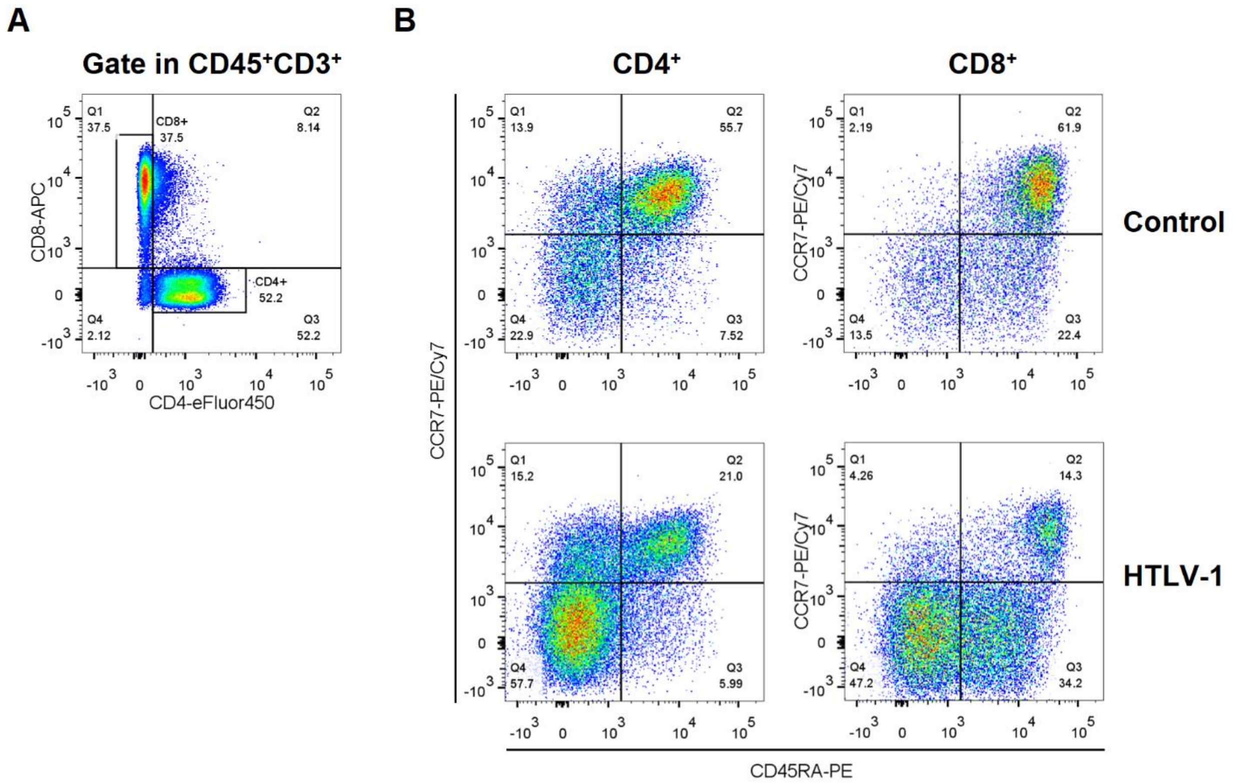

Figure S2. Maturation of CD4⁺ and CD8⁺ T-cells. HTLV-1-infected and control HIS-NSG mice were euthanized at 30 days and 60 days from inoculation. Blood, spleen and mesenteric lymph nodes (MLN) were collected, and the cells stained for flow cytometry analysis. **(A)** CD4⁺ and CD8⁺ T-cells were gated within human T-cells (CD45⁺CD3⁺ cells), and **(B)** the expression of CD45RA and CCR7 was evaluated. Cells were divided into naïve (Q2: CD45RA⁺CCR7⁺), central memory (Q1: CD45RA⁻CCR7⁺), effector memory (Q4: CD45RA⁻CCR7⁻), and terminal effector (Q3: CD45RA⁺CCR7⁻) cells.

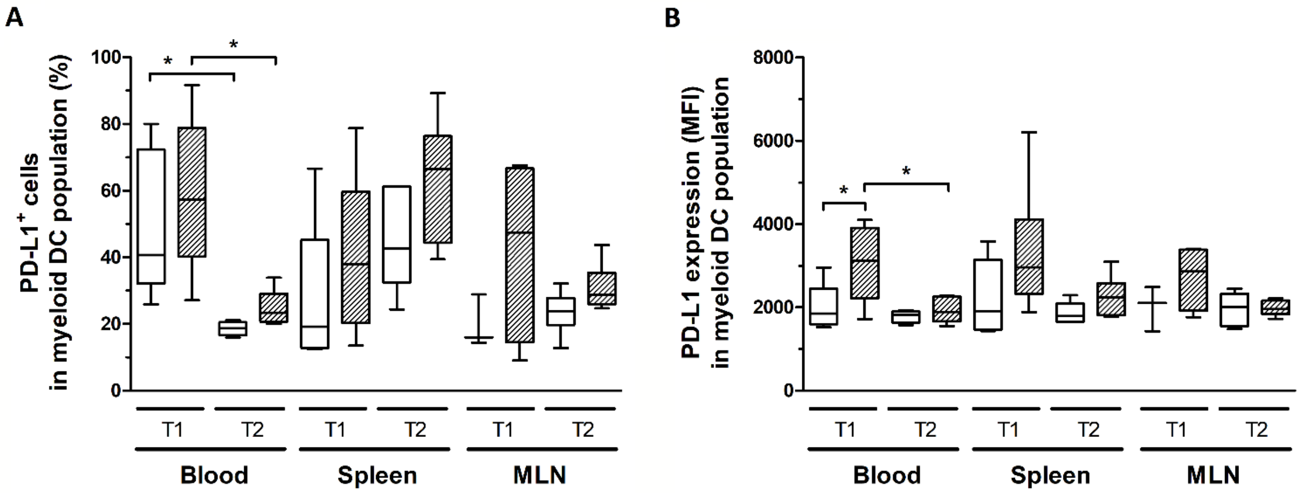

Figure S3. Expression of PD-L1 in myeloid dendritic cells (mDCs). Blood, spleen and mesenteric lymph nodes (MLN) from HTLV-1-infected (dashed box) and control HIS-NSG mice (white box) were collected at 30 days and 60 days from inoculation. **(A)** Frequency of PD-L1⁺ mDCs, which were identified as HLA-DR⁺CD11c⁺CD14⁻ cells within the gate of human CD45⁺ cells. **(B)** Mean fluorescence intensity (MFI) values were determined in the population of PD-L1⁺ mDCs. Statistical analysis was performed with Student's *t*-test (*, $p<0.05$). Control groups 30d and 60d: n=7; HTLV-1-infected group 30d: n=8; HTLV-1-infected group 60d: n=6.
